# Determination of N-acylhomoserine lactones of *Pseudomonas aeruginosa* in clinical samples from dogs with otitis externa

**DOI:** 10.1186/s12917-016-0843-0

**Published:** 2016-10-18

**Authors:** Darja Kušar, Karin Šrimpf, Petra Isaković, Lina Kalšek, Javid Hosseini, Irena Zdovc, Tina Kotnik, Modest Vengušt, Gabrijela Tavčar-Kalcher

**Affiliations:** Veterinary Faculty, University of Ljubljana, Gerbičeva 60, SI-1115 Ljubljana, Slovenia

**Keywords:** Dogs, Otitis externa, Clinical samples, *Pseudomonas aeruginosa*, Quorum sensing, N-acylhomoserine lactones, Liquid chromatography-tandem mass spectrometry, Validation

## Abstract

**Background:**

Bacterial intercellular communication, called quorum sensing, takes place via the production and collective response to signal molecules. In Gram-negative bacteria, like *Pseudomonas aeruginosa*, these signaling molecules are N-acylhomoserine lactones (AHLs). *P. aeruginosa* is a common cause of inflammation of the ear canal (otitis externa) in dogs. It employs quorum sensing to coordinate the expression of host tissue-damaging factors, which are largely responsible for its virulence. The treatment of *P. aeruginosa*-associated otitis is challenging due to a high intrinsic resistance of *P. aeruginosa* to several antibiotics.

Attenuation of quorum sensing signals to inhibit bacterial virulence is a novel strategy for the treatment of resistant bacterial pathogens, including *P. aeruginosa*. Therefore, it is important to recognize and define quorum sensing signal molecules in clinical samples. To date, there are no reports on determination of AHLs in the veterinary clinical samples. The purpose of this study was to validate an analytical procedure for determination of the concentration of AHLs in the ear rinses from dogs with *P. aeruginosa*-associated otitis externa.

Samples were obtained with rinsing the ear canals with physiological saline solution. For validation, samples from healthy dogs were spiked with none or different known amounts of the selected AHLs. With the validated procedure, AHLs were analyzed in the samples taken in weekly intervals from two dogs, receiving a standard treatment for *P. aeruginosa*-associated otitis externa.

**Results:**

Validation proved that the procedure enables quantification of AHLs in non-clinical and clinical samples. In addition, a time dependent reduction of AHL concentration was detected for the treated dogs.

**Conclusions:**

Our results indicate that liquid chromatography coupled with tandem mass spectrometry (LC-MS/MS) is superior in detecting AHLs compared to other chromatographic techniques. This is the first report on determination of AHLs in the clinical samples of veterinary importance. The analytical procedure described in this paper is capable of supporting novel antimicrobial strategies, which target quorum sensing.

## Background

*Pseudomonas aeruginosa* is an important opportunistic pathogen of humans and animals, causing severe infections when the immune system is compromised [1, 2], or after long-lasting antibiotic treatments, injuries and medical procedures [3, 4]. *P. aeruginosa* is a Gram-negative bacterium ubiquitously present in the water and soil. It is naturally resistant to penicillin and aminopenicillins, and to the first and second generations of cephalosporins. It is usually susceptible to aminoglycosides, fluoroquinolones, lipopeptides, ureidopenicillins, carboxypenicillins, carbapenems, and cephalosporins of the third generation [5]. The resistance of *P. aeruginosa*, however, can rapidly develop against any antibiotic [5].

Behavior of bacteria is coordinated, at the level of microbial community, by their gene expression at the intra-species level or less commonly between species. Such coordination of communal behavior is controlled through the production and detection of signal molecules or autoinducers, which is called quorum sensing (QS) [6–10]. The QS system defines the expression of various bacterial phenotypes, depending on the local density of bacterial population, including the virulence factors, biofilm formation and resistance to antibiotics [11]. It is associated with pathogenesis of various diseases in humans and animals, and has a clinically evident effect in human cystic fibrosis (CF) [12], endocarditis and osteomyelitis in rabbits [13, 14], bovine mastitis [15], gastrointestinal diseases [9], and ear canal infections, dermatitis and other secondary infections in dogs [16].

The secondary factors, including *Malassezia* spp. (yeasts), and bacteria of the genera *Staphylococcus*, *Proteus*, *Streptococcus* and *Pseudomonas*, often cause complications to the inflammation of the ear canal (otitis externa, OE) in dogs [17, 18]. Involvement of *P. aeruginosa* in OE (*P. aeruginosa*-associated OE, *Pa*OE) increases the morbidity, prolongs the treatment, and reduces the possibility of a favorable outcome [19, 20]. The ability of *P. aeruginosa* to produce biofilms and synchronize its virulence through QS is very important for the conservation and/or perpetuation of infection [21]. The long and narrow ear canal of dogs represents a very suitable environment for *P. aeruginosa* colonization and the subsequent activation of its QS [7].

One or several signal molecules may be involved in QS. In Gram-negative bacteria, the most common are N-acylhomoserine lactones (AHLs or AI-1) [9, 10, 22, 23]. *P. aeruginosa* QS system shows a hierarchical arrangement with *las* system controlling *rhl* [6–8, 24–28]. It mainly produces N-(3-oxododecanoyl)-L-homoserine lactone (3-oxo-C12-HSL) and N-butanoyl-L-homoserine lactone (C4-HSL), whereas production of N-hexanoyl-L-homoserine lactone (C6-HSL) and shorter 3-oxo-HSLs (3-oxo-C6-HSL, 3-oxo-C8-HSL and 3-oxo-C10-HSL) is less abundant [29, 30]. It was shown that 3-oxo-C12-HSL has the most critical role, as it regulates the expression of several genes involved in activation of the subordinate *rhl* system, where C4-HSL is the most significant autoinducing molecule [6]. In addition to its signalling function, 3-oxo-C12-HSL also exerts antibacterial activities against some other pathogens [31] and a direct immunomodulatory effect on the mammal host [32], itself functioning as a virulence determinant [33] and contributing to the establishment of chronic infections [34]. Under some growth conditions, activation of the *las* system does not precede the activation of *rhl*, but just the opposite, indicating the environmental dependence of their hierarchy [35]. There are various layers of sophistication to *P. aeruginosa* AHL-dependent QS [34, 36] and approximately 10 % of *P. aeruginosa* genome is directly or indirectly regulated by this multi-signal QS system [37]. Some genes are *las* specific, some *rhl*, and others respond to both 3-oxo-C12-HSL and C4-HSL [37]. In addition, at least four QS signalling mechanisms were reported to comprise *P. aeruginosa* QS network, being modulated in accordance to the environmental cues, also of host origin, influencing the virulence phenotypes of *P. aeruginosa* [36].

Detection of signal molecules associated with QS could serve as an indicator for the severity of infection, success of a treatment, and to estimate the outcome of disease [22]. Disruption of QS, called quorum quenching (QQ) [38], could represent a therapeutic tool, complementing or substituting the antibiotic treatment, which would reduce the risk for the development of antibiotic resistance [39]. It was shown that QQ can be achieved through the enzymatic destruction of QS signals, development of antibodies to QS signal molecules, or via agents which block QS [40]. The inhibitors of QS would reduce bacterial pathogenicity, also by increasing the susceptibility of existing biofilms to antibiotics or phagocytosis, and increase effectiveness of the host immune system [39, 41, 42]. Identifying molecules involved in QS in the clinical samples is, therefore, of great importance.

Several reports describing chemical analytical methods for the identification and quantification of selected AHLs are available. The use of a thin layer chromatography [7, 12]; gas chromatography with mass spectrometry (GC-MS) [43, 44]; high-performance liquid chromatography (HPLC) with UV detection [6], mass spectrometry (MS) [12], or coupled with tandem MS (MS/MS) [22, 45]; nuclear magnetic resonance (NMR) [26]; and colorimetry [46] was reported. In addition, a bacterial biosensor system for the detection of AHLs was also described [9]. Although a number of analytical methods were developed, only a few are applicable for determination of low AHL concentrations in clinical samples [22]. Determination of AHLs in physiological samples or complex biological matrices is more complicated than in bacterial culture supernatants due to the interference from matrix, causing most of the analytical problems [7, 9, 12, 22]. The mechanisms behind this phenomenon, commonly referred to as matrix effects and leading to signal suppression or enhancement, are still under investigation due to the great variety of available matrices and the unpredictable effect they might have on the final result [47]. Hence, data defining pathophysiological concentrations of AHLs in the clinical samples have not been often reported. In picomolar to micromolar concentrations, AHLs were discovered in the sputum [7, 12], mucopurulent secretions of respiratory tract [48], lung tissue of patients with CF [49], and saliva and faeces of individuals with gastrointestinal disorders [22]. To the best of our knowledge, no reports were published regarding detection of QS signal molecules in veterinary clinical samples.

In the present work, the analytical procedure for determination of AHLs of *P. aeruginosa* from canine OE clinical samples was introduced and validated. It consisted of the extraction of AHLs (3-oxo-C12-HSL, C4-HSL and C6-HSL) from the ear rinses and their quantification using the liquid chromatography with tandem mass spectrometry (LC-MS/MS).

## Methods

### Reagents

Solid standard substances C4-HSL, C6-HSL and 3-oxo-C12-HSL with certified purity of 97 % (Sigma-Aldrich, St. Louis, MO, USA) were used. Stock standard solutions of individual AHLs with the concentration of 1 mg/ml (C4-HSL, C6-HSL) and 0.91 mg/ml (3-oxo-C12-HSL) were prepared in methanol. Mixed working standard solutions for preparing the calibration curve containing all the three selected AHL signal molecules, each at the concentration from approx. 1 to 60 ng/ml, were prepared in a mixture of methanol and deionized water (35 + 65). Dichloromethane used for the extraction of AHLs and methanol used for the mobile phase were of analytical and chromatography grade purity, respectively (Merck, Darmstadt, Germany). As the mobile phase, a gradient mixing of methanol and deionized water containing 0.1 % formic acid (Merck) was used. For the rinsing of canine ear canals, saline solution (0.9 % NaCl) was employed.

### Sample collection and preparation

Samples were obtained by rinsing the ear canals of dogs with saline solution (30 ml). The collected samples were adjusted to the final volume of 30 ml by sterile saline, centrifuged at 3100 × g for 30 min at 4 °C, and the supernatants were stored at −80 °C till extraction.

For validation, the ear rinses from healthy dogs spiked with known amounts of C4-HSL, C6-HSL and 3-oxo-C12-HSL were used. None of the samples contained AHLs in detectable concentrations prior to spiking. An aliquot of 0.320 ml of the mixed working standard solution containing AHLs in concentrations of approx. 60 ng/ml (61.5 ng/ml of C4-HSL and C6-HSL, 56.9 ng/ml of 3-oxo-C12-HSL) was added to 10 ml of the supernatant, giving the final concentrations of approx. 2 ng/ml (1.97 ng/ml of C4-HSL and C6-HSL, 1.82 ng/ml of 3-oxo-C12-HSL). Thus, a 10-ml sample contained approx. 20 ng of each AHL (19.7 ng of C4-HSL and C6-HSL, 18.2 ng of 3-oxo-C12-HSL).

To test the validated analytical procedure with clinical samples, the ear rinses of two *Pa*OE dogs, collected weekly during the treatment, were used. Samples were taken during a scheduled veterinary checkup at the primary clinic or at the university clinic. As a control, the ear rinse from a healthy dog was employed.

### Analytical procedure

For the extraction of AHLs from the ear rinses, a procedure used for determination of AHLs in sputum [7, 12] was modified. One third (10 ml) of a 30-ml sample was extracted three times with 10-ml portions of dichloromethane. The combined extracts were evaporated to dryness at 60 °C using the vacuum evaporator (Syncore Reactor R-48, Büchi, Flawil, Switzerland). The residue was dissolved in 1 ml of the mixture of methanol and deionized water (35 + 65).

The particular AHLs were determined by LC-MS/MS at conditions described elsewhere [22, 45]. In brief, elution of the column was performed with a mixture of methanol–water. The mixing was isocratic for 4 min (35 % methanol, 65 % deionized water), followed by a linear gradient of methanol concentration from 35 to 95 % in 4 min and then back to 35 % in 1 min. The following 2 min, the column was washed again with the mixture of methanol and water (35 + 65). The mobile phase flow rate was 0.25 ml/min, injection volume 10 μl, and column temperature 30 °C. Detection with MS was performed in the positive ion mode. Voltage at the capillary was 0.4 kV. The monitored ions and other parameters are given in Table 1. Measurements were performed with the liquid chromatography system Acquity (Waters, Milford, MA, USA) equipped with an analytical column ZORBAX Eclipse C18, Rapid Resolution HD, 1.8 μm, 2.1 × 100 mm (Agilent, Santa Clara, CA, USA) and mass selective detector Xevo TQ MS (Waters).

**Table 1:** LC-MS/MS parameters

AHL	Retention time [min]	Precursor ion m/z^a^	Product ion m/z^a^	Cone voltage [V]	Collision cell voltage [V]
C4-HSL	1.99	172.096	101.980	18	10
		172.096	144.073	18	8
C6-HSL	4.76 (4.77)	200.160	101.984	18	10
		200.160	98.986	18	6
3-oxo-C12-HSL	9.16	298.266	102.038	26	12
		298.266	197.138	26	14

^a^m/z: mass-to-charge ratio

### Validation procedure

To perform the linearity test, and to determine the limit of detection (LOD) and the limit of quantification (LOQ), the mixed working standard solutions of AHLs at concentrations from approx. 1 to 60 ng/ml were used, corresponding to AHL concentrations of approx. 0.1 to 6 ng/ml in the ear rinses. The estimation of LOD and LOQ was based on visual evaluation, testing concentrations in the target range expected for AHLs in clinical samples. The LOQ estimate was defined as the lowest amount or concentration of an analyte that is reasonably achievable according to the target range and should be regarded as the reporting limit; LOD was estimated as one third (30 %) of LOQ [50].

The repeatability of results was tested by analyzing five replicates of the ear rinse sample with the addition of C4-HSL, C6-HSL and 3-oxo-C12-HSL at 1.97 ng/ml (C4-HSL, C6-HSL) and 1.82 ng/ml (3-oxo-C12-HSL). The analyses were performed at the same day and by the same operator. For testing the within-laboratory reproducibility, five replicates of the sample were prepared for two more times by two operators at two different days. The results obtained from these experiments were also used for determination of the recovery.

### Statistical procedures

The mean $\left(\overline{x}\right)$ for each AHL was calculated. The repeatability and the within-laboratory reproducibility were expressed by standard deviation (*s*_*r*_ and *s*_*R*_, respectively) and relative standard deviation (*RSD*_*r*_ and *RSD*_*R*_, respectively). The *RSD*_*R*_ values were compared to the values calculated from the Horwitz equation (*RSD* = 2^(1 − 0.5 log *C*)^), taken as a reference. The recovery was calculated according to the equation $rec\left(\%\right)=100\frac{\overline{x}}{a}$, where *a* represents the spiked concentration of the analyte in the sample [50, 51].

### Culture examination

Prior to rinsing the ears of *Pa*OE affected dogs with sterile saline, cotton-swab samples were taken from the ears for bacteriological examination. Samples were inoculated onto blood agar plates (Columbia agar; Oxoid LTD, Basingstoke, Hampshire, England) supplemented with 5 % sheep blood and incubated aerobically at 37 °C for 48 ± 2 h. After 24-h and 48-h incubation, plates were examined for the growth of *Pseudomonas* spp. and other pathogenic bacteria. Colonies morphologically consistent with *P. aeruginosa* were subcultured on fresh blood agar plates for subsequent identification. Bacterial isolates were identified using the methods described by Quinn and colleagues [52]; biochemical characteristics of the isolates were determined using the commercial kit Api 20 NE (BioMérieux, France) according to the manufacturer’s instructions.

## Results

**Fig. 1:**
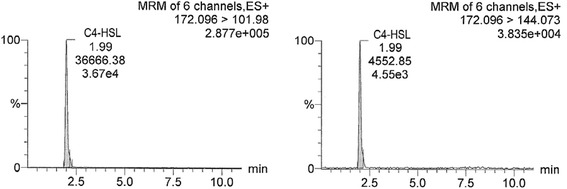
LC-MS/MS chromatogram of C4-HSL (N-butanoyl-L-homoserine lactone) standard solution with the concentration of 19.7 ng/ml. Transitions 172.096 > 101.98 and 172.096 > 144.073 are shown. The x-axis represents retention time (min) and y-axis the relative intensity of signal (relative to the highest peak in chromatogram, %)

**Fig. 2:**
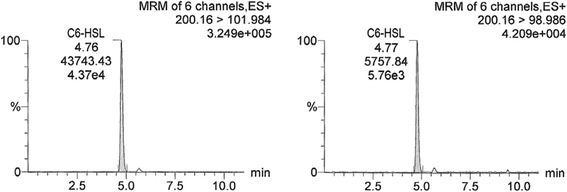
LC-MS/MS chromatogram of C6-HSL (N-hexanoyl-L-homoserine lactone) standard solution with the concentration of 19.7 ng/ml. Transitions 200.16 > 101.984 and 200.16 > 98.986 are shown. Description of the axes as in Fig. 1

**Fig. 3:**
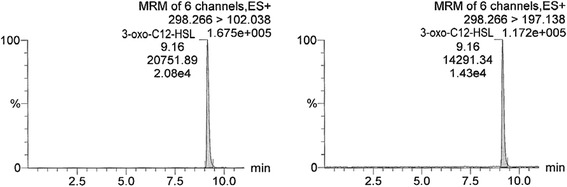
LC-MS/MS chromatogram of 3-oxo-C12-HSL (N-(3-oxododecanoyl)-L-homoserine lactone) standard solution with the concentration of 18.2 ng/ml. Transitions 298.266 > 102.038 and 298.266 > 197.138 are shown. Description of the axes as in Fig. 1

In Figs. 1, 2 and 3, chromatograms of individual AHL standard solutions C4-HSL (19.7 ng/ml), C6-HSL (19.7 ng/ml) and 3-oxo-C12-HSL (18.2 ng/ml) are presented, respectively. Retention times of C4-HSL, C6-HSL and 3-oxo-C12-HSL were 1.99, 4.76 (4.77) and 9.16 min, respectively (Figs. 1, 2 and 3, Table 1). For each AHL, two transitions of the precursor to product (fragment) ion are shown (e.g., for C4-HSL 172.096 to 101.980 and 172.096 to 144.073). In the figures, the common display of LC-MS/MS chromatograms, with the x-axis showing time and y-axis the relative signal intensity (relative to the highest peak in chromatogram, in %), was adopted.

Equations describing the calibration lines were *y* = 2027*x* + 708 (C4-HSL), *y* = 2603*x* + 2039 (C6-HSL) and *y* = 1594*x* + 204 (3-oxo-C12-HSL). Regression coefficients of the calibration curves were 0.999, 0.998 and 0.999, respectively. Based on the responses obtained, the lowest calibration point (1 ng/ml – or 0.1 ng/ml when referring to 10-ml volumes) was estimated as the lowest quantifiable concentration. Therefore, LOQ for 10-ml samples was estimated to 0.1 ng/ml (1 ng per 10-ml sample) and LOD accordingly lower to 0.03 ng/ml (0.3 ng per 10-ml sample). Expressed in molar concentrations, LOD and LOQ were approx. 175 pM and 584 pM for C4-HSL (with molar mass of 171.2 g/mol), 151 pM and 502 pM for C6-HSL (199.2 g/mol), and 101 pM and 336 pM for 3-oxo-C12-HSL (297.4 g/mol), respectively. Thus, with LOD in the range of 100–200 pM and LOQ in 300–600 pM, LC-MS/MS was confirmed suitable for the purpose as picomolar to micromolar concentrations were reported for clinical samples [7, 12, 22, 48, 49].

In Tables 2 and 3, parameters of the repeatability and reproducibility test are given. Recoveries, calculated from the results obtained under the within-laboratory reproducibility conditions, were 54 , 89 and 79 % for C4-HSL, C6-HSL and 3-oxo-C12-HSL, respectively. Before calculations, the outliers were excluded using the Grubbs statistical test [50].

**Table 2:** Parameters obtained within the repeatability test

Replicate	C4-HSL	C6-HSL	3-oxo-C12-HSL
Average mass [ng] in 10-ml sample	11.3	19.9	15.6
*s*_*r*_ [ng]	5.7	2.2	2.8
*RSD*_*r*_ [%]	50.2	11	18

**Table 3:** Parameters obtained within the reproducibility test

Replicate	C4-HSL	C6-HSL	3-oxo-C12-HSL
Average mass [ng] in 10-ml sample	10.6	17.5	14.3
*s*_*R*_ [ng]	3.7	3.8	2.9
*RSD*_*R*_ [%]	35	22	20
Recovery [%]	54	89	79

**Fig. 4:**
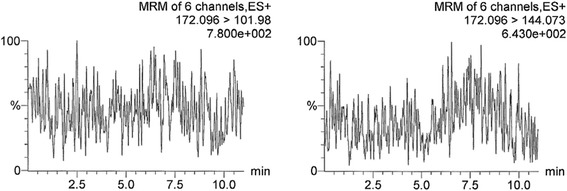
LC-MS/MS chromatogram of the ear rinse sample from a healthy dog, i.e., the representative chromatogram of a blank matrix. The noisy line reflects only the negative background, as the y-axis scale was normalized to the largest peak of 7.800 × 10^2^ units. Description of the axes as in Fig. 1

**Fig. 5:**
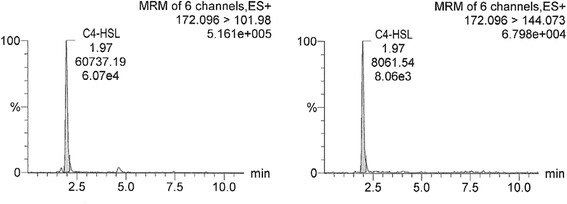
LC-MS/MS chromatogram of the ear rinse sample from a dog with *Pa*OE (*P. aeruginosa*-associated OE), i.e., the representative chromatogram of a positive sample containing C4-HSL (N-butanoyl-L-homoserine lactone). The y-axis scale was normalized to the peak of 5.161 × 10^5^ units. Chromatograms for other clinical samples appeared similar when regarding detection of individual AHLs, due to the presentation of analyte quantity in relative units and the characteristic analyte retention times. Description of the axes as in Fig. 1

When the analytical procedure for determination of C4-HSL, C6-HSL and 3-oxo-C12-HSL was successfully validated, the ear rinses (clinical samples) obtained from a healthy dog (negative control) and two dogs with *Pa*OE (positive samples) were analyzed. In Figs. 4 and 5, a chromatogram of the negative control and an example of C4-HSL chromatogram belonging to a positive sample from the *Pa*OE diseased dog are shown, respectively. The positive-sample chromatogram clearly shows a peak belonging to C4-HSL (Fig. 5), which is missing in the negative-control chromatogram (Fig. 4). It should be recalled that the y-axis scales are in relative units (normalized to the largest peak), resulting in the jagged line reflecting only the background noise in Fig. 4. The largest peak was 7.800 × 10^2^ units in Fig. 4 and 5.161 × 10^5^ units in Fig. 5, therefore the signal was approx. 1000-times higher in the positive sample than in the negative.

In Table 4, the total masses of C4-HSL, C6-HSL and 3-oxo-C12-HSL determined in the ear rinses of two dogs with *Pa*OE collected at weekly intervals during the treatment (week 0, 1, 2, 3, 4, 6 and 8) are given. The contents of C6-HSL and 3-oxo-C12-HSL were distinctly lower than of C4-HSL, making useful only the measurements of C4-HSL contents. In the first dog, the content of C6-HSL was much lower than of C4-HSL, but roughly correlated to fluctuations of C4-HSL; C6-HSL masses were close to LOQ (1 ng per 10-ml sample; week 0–3), but also below LOQ (week 8) or even LOD (0.3 ng per 10-ml sample; week 4 and 6). Surprisingly, the content of 3-oxo-C12-HSL was always below LOD. In the second dog, the contents of C6-HSL and 3-oxo-C12-HSL were below LOD but reached LOQ (C6-HSL) or LOD (3-oxo-C12-HSL) in the third and fourth week, which was in congruence with the increased C4-HSL levels.

**Table 4:** Total masses of AHLs in the ear lavages of dogs with *Pa*OE (*Pseudomonas aeruginosa*-associated OE), sampled in the course of treatment

Day	Week	1st dog				
		AHL mass [ng]			*Culture examination* ^*a*^	
		C4-HSL	C6-HSL	3-oxo-C12-HSL	*P. aeruginosa*	*Other bacteria*
0	0	33.7	1.1	<0.3	+	–
7	1	26.7	1.1	<0.3	+	–
14	2	29.4	1.2	<0.3	+	–
21	3	15.3	1.2	<0.3	+	–
28	4	1.3	<0.3	<0.3	+	–
42	6	2.7	<0.3	<0.3	+ (two strains^b^)	–
56	8	20.7	<1	<0.3	+ (two strains)	–
Day	Week	2nd dog				
		AHL mass [ng]			*Culture examination*	
		C4-HSL	C6-HSL	3-oxo-C12-HSL	*P. aeruginosa*	*Other bacteria*
0	0	<0.3	<0.3	<0.3	+	–
7	1	8.2	<0.3	<0.3	+	*Proteus mirabilis*
14	2	1.9	<0.3	<0.3	+ (two strains)	–
21	3	21.2	1.6	<1	+ (two strains)	*Proteus mirabilis*
28	4	32.4	1.5	<1	+ (two strains)	*Proteus mirabilis*
42	6	3.1	<0.3	<0.3	+ (two strains)	–
56	8	3.4	<0.3	<0.3	+	–

^a^Results of culture examination are given for better understanding and are explained in the text

^b^Indicating a secondary infection with a different *P. aeruginosa* strain, deteriorating the clinical condition of dogs and causing the increase of AHL content in the infected ear canal

In the first dog, the content of C4-HSL decreased in the first month of treatment, but then slightly increased in the sixth week and markedly in the eighth week of treatment (Table 4). At that time (week 6 and 8), a secondary infection of the ear canal was also observed by culture examination, showing the presence of two different *P. aeruginosa* strains. In the second dog, the disease management started with C4-HSL below LOD (day 0), but its content increased after the first week of treatment (Table 4). The time coincided with isolation of *Proteus mirabilis* from the ear swab. In the second week of treatment, the content of C4-HSL decreased and *P. mirabilis* was no longer detected by culture examination. In the third and fourth week, however, a marked increase of C4-HSL content could be observed, with the appearance of additional *P. aeruginosa* strain (already in week 2) and the reappearance of *P. mirabilis*. Later, in the second month of the treatment, the content of C4-HSL decreased and the secondary infection was resolved (week 6 and 8).

## Discussion

The present study demonstrated that AHL signal molecules can be quantified in the ear rinses of *Pa*OE affected dogs using LC-MS/MS. This method proved superior when compared to other chromatographic techniques that were tested in the scope of our preliminary validation experiments: GC-MS [43, 44] and HPLC with UV detection [6]. Namely, of the three methods selected for determination of AHLs in OE clinical samples only LC-MS/MS enabled determination of signal molecules at target concentrations. Two major (3-oxo-C12-HSL, C4-HSL) and one minor (C6-HSL) AHL of *P. aeruginosa* were monitored. Identification of AHLs in clinical samples can have an important diagnostic, therapeutic and prognostic value in human and veterinary medicine.

The detection and quantification limits of the analytical procedure were low enough (LOD: 0.03 ng/ml; LOQ: 0.1 ng/ml) to enable determination of C4-HSL, C6-HSL and 3-oxo-C12-HSL in the ear rinses from dogs with *Pa*OE. The linearity of the procedure was confirmed with the regression coefficient of the calibration curve higher than 0.995. The precision, repeatability and the within-laboratory reproducibility, of the procedure was expressed by standard deviations *s* [ng] and relative standard deviations *RSD* [%]. Two outliers were eliminated based on the Grubbs test [50], both belonging to the 3-oxo-C12-HSL measurements from the same day of validation. The resulting *RSD*_*R*_ values for C4-HSL (35 %), C6-HSL (22 %) and 3-oxo-C12-HSL (20 %) were compared to the reference values derived from the Horwitz equation: for the tested concentration of 2 ng/ml, the reference *RSD*_*R*_ value was 41 % [50, 51]. Since the obtained *RSD*_*R*_ values were below 41 %, the reproducibility was considered satisfactory.

The recoveries of C4-HSL, C6-HSL and 3-oxo-C12-HSL at the concentration of 2 ng/ml were 54, 89 and 79 %, respectively. Regarding requirements for the performance of analytical procedures (e.g., Decision 2002/657/EC [51]), the recoveries of C6-HSL and 3-oxo-C12-HSL were considered satisfactory; whereas the recovery of C4-HSL (54 %) was suboptimal (it should be 70–110 %). Similar recovery values were reported previously [7]. The suboptimal recovery of C4-HSL should not be considered a significant limiting factor, because the analytical procedure was primarily used for monitoring the changes in AHL concentrations with time and not to determine their absolute values. However, the obtained recovery values can be used for the correction of results to achieve a good approximation of the actual contents in a sample whenever appropriate.

Sample sediments, which were obtained after the initial centrifuging of the ear rinses, were tested for residual levels of AHLs prior to validation. This was performed to verify the predominating distribution of analytes into the liquid phase. Sediment analysis was the same as implemented for the supernatants. The quantities of individual AHLs were approximately 20-times lower in the sediments than in their respective supernatants (data not shown), indicating the residual level of less than 5 %. Therefore, only supernatants were used for further analysis.

Samples for the present study were collected from two dogs with *Pa*OE, which was confirmed with the routine bacteriological analyses. The treatment regimen was standard [53–58] and included regular ear irrigations with topical saline flush and local administration of Tris-EDTA/chlorhexidine (0.15 % solution; Otodin, Industria Chimica Fine ICF, Palazzo Pignano, Italy) once daily. Namely, the antiseptic solution Tris-EDTA/chlorhexidine was proven beneficial and safe for the *Pa*OE diseased dogs with unlikely selection for bacterial resistance which would predispose the chronic character of the disease [55–58]. A systemic antibiotic (enrofloxacin) treatment was only employed after clinical condition of dogs deteriorated. To avoid confusion, the state of clinical condition should not be directly deduced from the results of culture examination or AHL content in the samples. Enrofloxacin was administered in the last 14 days of treatment to the first dog and in the first 14 days to the second.

Regarding the total masses of AHLs in the ear lavages of dogs with *Pa*OE, the content of C6-HSL was in general much lower than of C4-HSL, but the fluctuations of these two roughly correlated. The content of C6-HSL was approximating LOQ or even LOD. Moreover, the content of 3-oxo-C12-HSL was always below LOQ or, in most cases, below LOD. According to the results, only C4-HSL measurements showed applicable value. In the first dog, results showed that the content of C4-HSL decreased in the first month of Tris-EDTA/chlorhexidine treatment in accordance to expectations [58], but increased again toward the end of the treatment (slightly in week 6 and markedly in week 8). The increase could be assigned to a secondary infection of the ear canal, which was confirmed by culture examination showing the presence of an additional *P. aeruginosa* strain.

In the second dog, the content of C4-HSL was below LOD at the beginning of the disease management, but increased after the first week of treatment. *P. mirabilis*, a Gram-negative opportunistic pathogen ubiquitously present in the environment and a common secondary factor of OE in dogs [17, 18], was also isolated from the ear canal. Since Gram-negative bacteria employ AHLs for quorum communication, cross-talk communication between the two species cannot be excluded. The inter-species communication was suggested previously between *P. aeruginosa* and *P. mirabilis*, since a variation of AHL QS system was observed in *P. mirabilis* with signaling molecules being structurally similar to AHLs [59]. Namely, in both the cell-free culture supernatants of *P. aeruginosa* and of *P. mirabilis*, compounds capable of activating the AHL biosensor were found, not belonging to AHLs but to structurally similar diketopiperazines (DKPs). In concentration-dependent manner, DKPs were able to activate the AHL biosensor, although their physiological role remains to be discovered, but also suggesting the existence of cross talk among bacterial signaling systems. However, the QS system in *Proteus* sp. is not completely understood and its signaling molecules are not well defined. Although signaling molecules with the structure identical to AHLs are not produced by *P. mirabilis* [60], it was shown that *P. mirabilis* reacts and changes the population features when AHLs produced by other Gram-negative bacteria are added to the culture media [61]. The content of C4-HSL in the second dog decreased in the second week of treatment, which coincided with the disappearance of *P. mirabilis* from the bacteriological sample. The marked increase in C4-HSL was detected in the third and fourth week, which coincided with an additional strain of *P. aeruginosa* and the reappearance of *P. mirabilis* in the bacteriological sample. After the fourth week, the C4-HSL content declined and *P. mirabilis* was no longer present. By the end of the treatment course, the additional strain of *P. aeruginosa* also disappeared.

No AHLs were detected in the negative control sample from a healthy dog. The analytical procedure described and validated in this study is, therefore, capable of monitoring changes in AHL concentration throughout the treatment regimen, reflecting the state of *Pseudomonas* infection.

Concerning 3-oxo-C12-HSL, low concentrations were detected in this study. Similar was observed previously in samples from human patients with CF [7]. Several, interconnected or independent, reasons for this analytical outcome are possible: *i*) 3-oxo-C12-HSL is not a major AHL present in the ear canals of *Pa*OE-diseased dogs or in clinical samples in general; *ii*) deficiency of one or more *P. aeruginosa* QS components leading to reduced synthesis/deployment of 3-oxo-C12-HSL; *iii*) the in-vitro results, which are presently the gold standard for understanding the biology of QS and AHLs, are different from the in-vivo behavior of *P. aeruginosa* QS; and *iv*) limitations of the membrane transport, which are selective to effusion of 3-oxo-C12-HSL from *P. aeruginosa* cytoplasm.

Regarding the results obtained in this study and previously for the samples from human CF patients [7], it is possible that 3-oxo-C12-HSL is not a major AHL present in clinical samples, disease-related or in general. One should not be misled by the terminology that the *rhl* system is ‘only’ subordinate to *las*, as we can speculate that *rhl* might be more adaptable to current conditions in its milieu or could support greater part of QS communication. A deficiency of one or more QS components of the *rhl* system was previously reported for *P. aeruginosa* canine OE isolates [62], and *P. aeruginosa* mutants with the impaired *las* were also obtained from clinical samples [63]. These QS deficient strains are regularly isolated from clinical or environmental samples and are called the ‘cheating’ individuals, profiting from the benefits of the cooperating population without contributing to the community [64]. Not all *P. aeruginosa* strains/isolates produce both C4-HSL and 3-oxo-C12-HSL [7, 63]. In addition to the QS deficiencies, the expression of QS is different in *P. aeruginosa* isolates from clinical samples or grown as biofilms when compared to those that are grown in laboratory as broth cultures in nutrient-rich media [12, 65]. Broth cultures are being intensively studied to explain the composition and hierarchy of bacterial QS systems. In the natural environments, however, biofilms are predominating over the planktonic growth [7, 12]. *P. aeruginosa* is notorious for its ability to form biofilms [10]. Isolated from CF sputum of human patients, *P. aeruginosa* produces C4-HSL in excess of 3-oxo-C12-HSL when grown in biofilms but not in broth cultures [65]. If the higher content of C4-HSL over 3-oxo-C12-HSL observed in the present study is due to variation in their production, *P. aeruginosa* from the ear canals of *Pa*OE-diseased dogs is also predominantly present as biofilms. Another reason for low concentrations of 3-oxo-C12-HSL in the studied clinical samples, in addition to QS deficiencies of *P. aeruginosa* strains and differential gene expression in biofilms, could be the fact that 3-oxo-C12-HSL requires an active membrane transport (pump) for its efflux from the cells to the surroundings, whereas C4-HSL diffusion has no such limitation and *P. aeruginosa* cells are freely permeable to C4-HSL [7, 66].

If *P. aeruginosa* is predominately growing in biofilms in the ear canals of *Pa*OE-diseased dogs, another explanation for the low amounts of 3-oxo-C12-HSL can be suggested. Namely, AHLs are restricted in diffusion by the extracellular matrix of biofilms and they reach adequate intracellular concentrations for induction of QS-dependent genes at lower values in comparison to planktonic growth phenotype [67]. Therefore, it is possible that less 3-oxo-C12-HSL is needed in biofilms for the activation of the subordinate *rhl* system, but more with planktonic phenotypes due to diffusion throughout the liquid milieu to reach concentrations high enough for the activation of *rhl*.

*P. aeruginosa* can adapt to different environments via its regulatory networks and multifunctional signal molecules [34, 36], which can be monitored using the methods such as the one reported in this study. Disrupting QS regulation is a valuable approach to develop new therapeutic strategies. Implementation of alternative therapies (QQ strategies) for *Pa*OE, or other *P. aeruginosa* infections, should have a beneficial impact on patients [68, 69]. Plants, for example, represent a large and unexplored pool of potentially QQ bioactive compounds [70, 71].

## Conclusions

The QS system is a defining part of the pathologies caused by *P. aeruginosa* and other bacteria. Detection and quantification of AHLs in clinical samples may provide an important diagnostic and prognostic tool to follow the disease progress, which has already been proven in human CF cases. It would also be possible to initiate and/or terminate, or evaluate, the success of antibiotic treatments in diseases of the *Pa*OE complexity. In this study, the LC-MS/MS analytical procedure for determination of selected *P. aeruginosa* AHLs (C4-HSL, C6-HSL, 3-oxo-C12-HSL) in clinical samples obtained from *Pa*OE-diseased dogs was introduced and successfully validated. For the clinical samples obtained from two *Pa*OE-diseased dogs, low content of 3-oxo-C12-HSL was observed with C4-HSL predominating. To explain the phenomenon, it was suggested, in addition to other hypotheses, that *P. aeruginosa* is growing in the infected ear canals organized in biofilms.

## Abbreviations

3-oxo-C12-HSL: N-(3-oxododecanoyl)-L-homoserine lactone
AHLs (or AI-1): N-acylhomoserine lactones
C4-HSL: N-butanoyl-L-homoserine lactone
C6-HSL: N-hexanoyl-L-homoserine lactone
CF: Cystic fibrosis
DKPs: Diketopiperazines
GC-MS: Gas chromatography with mass spectrometry
HPLC: High-performance liquid chromatography
LC-MS/MS: Liquid chromatography with tandem mass spectrometry
LOD: Limit of detection
LOQ: Limit of quantification
MS: Mass spectrometry
MS/MS: Tandem mass spectrometry
NMR: Nuclear magnetic resonance
OE: Otitis externa [inflammation of the external ear canal]
*Pa*OE: *Pseudomonas aeruginosa*-associated OE
QQ: Quorum quenching
QS: Quorum sensing
*rec*: recovery
*RSD*: Relative standard deviation (*RSD*_*r*_ for repeatability, *RSD*_*R*_ for reproducibility)
*s*: standard deviation (*s*_*r*_ for repeatability, *s*_*R*_ for reproducibility)
